# Chinese Herbal Medicines Facilitate the Control of Chemotherapy-Induced Side Effects in Colorectal Cancer: Progress and Perspective

**DOI:** 10.3389/fphar.2018.01442

**Published:** 2018-12-07

**Authors:** Dongmei Chen, Jun Zhao, Weihong Cong

**Affiliations:** ^1^Xiyuan Hospital of China Academy of Chinese Medical Sciences, Beijing, China; ^2^Graduate School, Beijing University of Chinese Medicine, Beijing, China; ^3^The University of Texas, MD Anderson Cancer Center, Houston, TX, United States; ^4^The Affiliated Hospital of Qingdao University, Qingdao, China

**Keywords:** Chinese herbal medicine, chemotherapy, side effects, colorectal cancer, review

## Abstract

Side effects, including nausea, vomiting, mucositis, peripheral neuropathy, and diarrhea, have been frequently reported in colorectal cancer (CRC) patients undergoing chemotherapy. Chinese Herbal Medicines (CHMs) display distinct clinical outcomes, as a result, they have been increasingly used as an adjuvant therapy to manage chemotherapy-induced side effects. In this review, we aim to intensively explore the molecular mechanisms of CHMs, underline the significance of CHMs in mitigating the side effects induced by chemotherapy, and examine the necessary studies required to understand the role of CHMs in alleviating chemotherapy-induced side effects. Specifically, ginger, *Astragali Radix*, and Liujunzi Decoction have been verified to ameliorate nausea and vomiting. Banxia Xiexin Decoction and Huangqin Decoction have been confirmed to be beneficial to mucositis and delayed-onset of diarrhea. Moreover, Niuche Shenqi Wan, Guilong Tongluo Decoction, Huangqi Guizhi Wuwu Decoction, and tumeric have been found to display potential therapeutic effects for preventing the genesis and development of peripheral neurotoxicity. These findings have further emphasized the pivotal role of CHMs in improving the outcomes of chemotherapy-induced side effects in CRC. Nonetheless, more molecular evidence is required to comprehensively understand and more appropriately apply CHMs in routine clinical practice for CRC.

## Introduction

Colorectal cancer (CRC) is one of the most common malignancies, with incidence ranking the third among males and females worldwide. In terms of mortality, CRC is in the third place and the fourth place in males and females, respectively (Siegel Rebecca et al., 2015). Chemotherapy is widely used and plays a pivotal role in improving the outcomes for CRC patients (Atreya and Neurath, 2008). Chemotherapy is effective against cancer, yet it is also associated with various side effects, including nausea, vomiting, diarrhea, oral mucositis, and neurotoxicity, which have thereby restricted its clinical application, lowered the quality of life of patients, and resulted in dose reductions, dose delays and even termination of chemotherapy (Wyatt et al., 2015).

Despite the advancement in various treatment strategies in managing chemotherapy-induced side effects, such as 5-HT3 receptor antagonist and dexamethasone for nausea and vomiting, calcium gluconate, magnesium sulfate and glutathione for attenuating peripheral neurotoxicity, and loperamide for irinotecan-induced diarrhea, these can hardly satisfy the requirements of patients undergoing chemotherapy (Mustian et al., 2011). Thus, the development of an alternative treatment to manage these side effects is required.

Chinese Herbal Medicines (CHMs) are combinations of herbs or a single herb. Most of them originate from ancient Chinese medical classics, for instance, “*Shang Han Lun*” or “*Jin Gui Yao Lue*.” Currently, CHMs are extensively prescribed to cancer patients. Some of these CHMs have been investigated and verified to be beneficial in alleviating chemotherapy-related side effects (Qi et al., 2010). In this review, we emphasize the current application of CHMs in dealing with the sides effects induced by chemotherapeutics, specifically those used in CRC, including 5-fluorouracil (5-Fu), capecitabine, oxaliplatin, and irinotecan.

## Neurotoxicity

Oxaliplatin, a third-generation derivative of platinum, is demonstrated to restrain tumor growth through its alkylating activity. It has been wildly used to treat various solid tumors and is often used to manage locally advanced and metastatic colon or rectal cancer (de Gramont et al., 2012). Its introduction has improved progression-free survival in patients (de Gramont et al., 2000). However, the development of neuropathy, one of the main side effects of oxaliplatin, is related to accumulated dose, which restricts its application (Lehky et al., 2004; Haller et al., 2011). Specifically, oxaliplatin is frequently used in combination with folinic acid (LV) and 5-Fu to formulate the FOLFOX regimen, which serves as an adjuvant therapy for CRC. Briefly, about 50% patients who have received cumulative dose of oxaliplatin (>1,000 mg/m^2^) developed chronic neuropathy (de Gramont et al., 2000; Souglakos et al., 2002). Paresthesia, dysesthesia, pain, and a glove-and-stocking distribution of sensory loss are the most common symptoms of oxaliplatin-induced neuropathy. Repeated drug administration frequently leads to an irreversible neuropathic syndrome or chronic neurotoxicity, which lowered the quality of life of patients (de Gramont et al., 2000). Nevertheless, the underlying mechanism by which oxaliplatin induces neurotoxicity remains unclear. It has been reported that oxaliplatin accumulation in the dorsal root ganglia affects the sensory nerve cell bodies, which can cause axonal hyperexcitability and repetitive discharges as a result of changes in the voltage-dependent sodium channels (Pasetto et al., 2006; Windebank Anthony and Grisold, 2008). Moreover, it was reported that the administration of calcium gluconate, magnesium sulfate, and glutathione can attenuate peripheral neurotoxicity (Gamelin et al., 2002, 2004; von Delius et al., 2007). However, an effective remedy for oxaliplatin-induced neurotoxicity has not yet been established. Fortunately, some CHMs or Chinese herbal extracts have shown efficacy in alleviating oxaliplatin-induced neurotoxicity.

### Astragali Radix

*Astragali Radix*, or Huangqi in Chinese, is one of the commonly prescribed herbs to tonify Qi according to the traditional Chinese medicine theory. Different *Astragali Radix* extracts have been shown to protect nervous tissue from oxaliplatin-induced nerve damage without affecting the anticancer effect of chemotherapy (Deng et al., 2016a). Particularly, 50% hydroalcoholic extract of *Astragali Radix* was shown to reduce oxaliplatin-induced cold hypersensitivity, completely block the onset of the pro-allodynia effect, and relieve neuro-damage-induced pain in oxaliplatin-treated rats (Di Cesare Mannelli et al., 2015). Probably, such protective effects can be attributed to the down-regulated expression of the activating transcription factor 3, a neuronal marker of nerve injury, in the dorsal root ganglia (Di Cesare Mannelli et al., 2015, 2017). Although currently available evidence supporting the ability of *Astragali Radix* to reduce neurotoxicity is based on animal studies, it remains promising to conduct clinical trials to further identify its efficacy in human beings.

### Tumeric

Curcumin is the main phenolic compound of turmeric (*Rhizoma Curcumae*). Compared with the controls, rats treated with curcumin were found to have attenuated oxaliplatin-induced neurotoxicity, which was achieved through reducing high plasma neurotensin and platinum accumulation in the sciatic nerve, as well as markedly improving the sciatic nerve histology (Al Moundhri et al., 2013). Curcumin was also shown to attenuate Fluoride-induced neurotoxicity by decreasing the lipid peroxidation and neurodegeneration in mice treated with Fluoride (Sharma et al., 2014). *In vitro*, curcumin was demonstrated to protect against cisplatin neurite outgrowth inhibition in PC12 cells, which was regarded as an indicator of the protective potential against neuropathy (Mendonça et al., 2013).

### Guilong Tongluo Decoction

Guilong Tongluo Decoction is a formula constituted by *Ramulus Cinnamomi, Astragali Radix*, Earthworm*, Carthamus Tinctorius, Radix Angelicae Sinensis, Ligusticum, Spatholobus, Radix Paeoniae Alba, Rhizoma Curcumae*, and *Glycyrrhizae Radix*. It has been shown in a randomized double-blind and placebo-controlled clinical trial that, compared with controls, Guilong Tongluo Decoction remarkably alleviated the development of grades 1–2 neurotoxicity after 6 cycles of adjuvant oxaliplatin-based chemotherapy in CRC patients (70.0 vs. 51.7%, *P* < 0.05) (Liu et al., 2013a). In addition, Guilong Tongluo Decoction also delayed the onset time of grades 1–4 neurotoxicity relative to the control group (9.4 vs. 6.5 weeks in the trial vs. control groups, *P* < 0.05) (Liu et al., 2013a). Most importantly, no statistically significant difference was found in tumor response rate between the trial and the control groups, indicating that Guilong Tongluo Decoction is a promising prescription for preventing oxaliplatin-induced neurotoxicity in CRC patients without reducing the efficacy of oxaliplatin-based chemotherapy. However, the underlying mechanisms need to be further investigated.

### Niuche Shenqi Wan

Niuche Shenqi Wan is a frequently used prescription in Asia. It is comprised of 10 different herbs, including *Rehmannia Viride Radix, Achyranthis Bidentatae Radix, Corni Fructus, Dioscorea Opposita Rhizoma, Plantaginis Semen, Alismatis Rhizoma, Moutan Cortex, Cinnamomi Cortex, Aconiti Lateralis Praeparata Radix*, and *Poria Alba*. It has been shown in a retrospective study to mitigate oxaliplatin-induced neurotoxicity in advanced or recurrent CRC patients (Kono et al., 2011). Additionally, as demonstrated in another prospective clinical trial, Niuche Shenqi Wan decreased the incidence and simultaneously delayed the development of grade 2 and above oxaliplatin-induced neurotoxicity in CRC after 8 cycles of oxaliplatin-based chemotherapy compared with the placebo control (39 vs. 51%). Specifically, the incidence of grade 3 oxaliplatin-induced neurotoxicity was 7 vs. 13%, respectively, in the two groups (Kono et al., 2013, 2015). Preclinical studies reported that the effect of Niuche Shenqi Wan in alleviating oxaliplatin-related neurotoxicity, especially cold hyperalgesia and mechanical allodynia, could be attributed to increased peripheral blood flow, stimulation of spinal kappa-opioid receptors, inhibition of oxidative stress or activation of the C fiber (Kono et al., 2015). Furthermore, studies also confirmed that Niuche Shenqi Wan didn't interfere the anti-tumor effect of oxaliplatin both *in vitro* and *in vivo* (Ushio et al., 2012). Taken together, these studies have provided strong support that Niuche Shenqi Wan could attenuate neurotoxicity caused by oxaliplatin via restoring the slowed blood flow, inhibiting oxidative stress and activating the C fiber.

### Wenluotong Decoction

Wenluotong Decoction is a prescription composed of *Epimedium Brevicornum Maxim, Geranium Wilfordii Maxim, Cinnamomum Cassia Presl*, and *Carthamus Tinctorius*. It has been used as an external therapy in China to manage chemotherapy-induced neuropathic pain. As shown in a clinical study, Wenluotong Decoction could dramatically alleviate chemotherapy-induced peripheral neurotoxicity (Noh et al., 2018). Consistently, such results were confirmed in the mouse model of oxaliplatin-induced neuropathy, and it was found to reverse both glial activation in the spinal dorsal horn and nociceptive sensitization of oxaliplatin-resultant chronic neuropathic pain in rats (Deng et al., 2016b). Wenluotong Decoction is the only reported external formula applied and investigated in attenuating oxaliplatin-induced neuropathy so far.

### Guizhi Jiazhufu Decoction

Guizhi Jiazhufu Decoction is composed of *Cinamomi Cortex, Aconiti Lateralis Praeparata Tuber, Zingiberis Rhizoma, Jujubae Fructus, Glycyrrhizae Radix*, and *Atracylodis Macrocephalae Rhizoma*. It has been shown to reduce neuropathy in metastatic CRC patients undergoing the FOLFOX regimen (Hosokawa et al., 2012). More animal studies need to be conducted to determine the underlying mechanisms of Guizhi Jiazhufu Decoction in attenuating chemotherapy-induced neuropathy.

### Shaoyao Gancao Decoction

Shaoyao Gancao Decoction, a prescription composed of *Paeonia Alba Radix* and *Glycyrrhizae Radix*, has been employed to prevent the onset of neuropathy. Its response rate was reported to be 65% in advanced CRC patients with oxaliplatin-related neuropathy (Hosokawa et al., 2012). It was demonstrated to attain its effect through suppressing transient receptor potential melastatin 8 (characterized as a cold-sensing) mRNA expression in the mouse dorsal root ganglia (Andoh et al., 2017).

### Huangqi Guizhi Wuwu Decoction

Huangqi Guizhi Wuwu Decoction is composed of *Astragali Radix, Cinnamomi Cortex, Radix Paeoniae Alba, Zingiberis Rhizoma*, and *Jujubae Fructus*. AC591 is a standardized extract from Huangqi Guizhi Wuwu Decoction, which has been reported to lower the incidence and relieve the severity of neurotoxicity in patients undergoing the FOLFOX regimen. The incidence of grades 1-2 neurotoxicity was reported to be 25% in the AC591 group, and no peripheral neurotoxicity of grades 3-4 was observed. However, in the non-AC591 treatment group, over 55% of patients suffered from grade 1 or 2 neurotoxicity, and 8.33% developed grade 3 or 4 neurotoxicity. Clearly, the severity of neurotoxicity in the AC591 treatment group was lower than that in the non-AC591 treatment group (25 vs. 63.89%, *P* < 0.01). Moreover, it was also discovered in that study that the mechanism of the neuroprotective effect of AC591 might depend on modulating multiple molecular targets and pathways involved in down-regulating inflammation and immune responses (Cheng et al., 2017). This study contained both clinical and preclinical investigations and pointed out that Huangqi Guizhi Wuwu Decoction might play a role in immune responses while exerting a neuroprotective effect. A systemic review further confirmed that Huangqi Guizhi Wuwu Decoction was beneficial to treat as well as prevent the onset of neurotoxicity (Jun et al., 2013).

It's interesting that herbal decoctions mentioned above are composed of different herbs and some of them may contain the same single herb. The function of the same single herb in different herbal decoctions may vary based on the “Jun-Chen-Zuo-Shi” theory of CHMs. In an herbal decoction, herbs regarded as “Jun” (emperor) is applied to treat the main cause of the disease. “Chen” (minister) is usually utilized to enhance the function of “Jun.” “Zuo” (adjuvant) plays a role in reducing the toxicity of other herbs and treating accompanying symptoms. “Shi” (courier) helps to guide or deliver the other herbs to the target organ. For instance, both Huangqi Guizhi Wuwu Decoction and Guilong Tongluo Decoction contain *Astragali Radix*. *Astragali Radix* is regarded as “Jun” in Huangqi Guizhi Wuwu Decoction. It is applied to treat qi deficiency, which is the main cause of the disease. However, *Astragali Radix* is regarded as “Chen” in Guilong Tongluo Decoction. Thus, qi deficiency may not be the main cause in Guilong Tongluo Decoction treated patients. In addition, both Shaoyao Gancao Decoction and Guizhi Jiazhufu Decoction contain *Glycyrrhizae Radix. Glycyrrhizae Radix* is used to enhance the effect of *Paeonia Alba Radix* in alleviating neuroglia in Shaoyao Gancao Decoction. However, *Glycyrrhizae Radix* in Guilong Tongluo Decoction acts as “Shi” and helps coordinating other herbs in the decoction. Thus, different herbal decoctions can be used to treat chemotherapy induced neurotoxicity due to different Chinese Medicine symptoms. On the other hand, the same herb may play different roles in different herbal decoctions.

## Oral Mucositis

Chemotherapeutics contribute to killing the rapidly proliferated cancer cells. However, most of them show no selectivity, leading to damage to rapidly regenerated normal tissues, such as gastrointestinal and immune cells (Kwon, 2016). Oral mucositis is a common side effect in CRC patients undergoing 5-Fu or capecitabine treatment (Curra et al., 2018). To be specific, the incidence of oral mucositis at various severity degrees was reported to be approximately 90% in CRC patients treated with 5-Fu (Saini et al., 2003). Approximately 20% CRC patients undergoing capecitabine-based regimens developed oral mucositis (Lee et al., 2009; Baird et al., 2011). Oral mucositis is usually associated with ulcerative or erosive lesions in the oral mucosa, which causes pain and greatly lower quality of life (Sonis et al., 2001). The mechanisms involved in oral mucositis include apoptosis of submucosal cells, activation of macrophages and transcription factors [for example, nuclear factor kappa B (NF-κB)], and elevation of pro-inflammatory cytokines [such as tumor necrosis factor (TNF-α), interleukin 1 beta (IL-1β), cyclooxygenase-2 (COX-2), and matrix metalloproteinases]. Among them, activation of the inflammatory cytokine cascade gives rise to tissue injury and cell death (Sonis, 2007).

### Banxia Xiexin Decoction

Banxia Xiexin Decoction has been widely used to attenuate chemotherapy-induced gastrointestinal toxicity, which is composed of *Pinellia Ternata, Scutellaria Baicalensis Radix, Panax Ginseng, Rhizoma Coptidis, Glycyrrhizae Radix, Zingiberis Rhizoma*, and *Jujubae Fructus*. It was reported that, compared with the placebo control, Banxia Xiexin Decoction treatment combined with chemotherapy could dramatically reduce the severity grade (≥2) and the duration of chemotherapy-induced oral mucositis (15 vs. 8 days, *P* < 0.05) in gastric cancer and CRC patients (Matsuda et al., 2015; Nishikawa et al., 2018).

Moreover, other Chinese prescriptions, such as Yinqiaosan Powder, Yunujian Decoction, and Qingying Decoction, have also been proven to be partially beneficial to chemotherapy-induced oral mucositis (Meyer-Hamme et al., 2013). *Lonicerae Flos*, one herb in these prescriptions, was found to suppress the expression of COX-2, IL-1, and IL-6 (Schröder et al., 2013). Additionally, *Glycyrrhizae Radix*, another important herb used in these prescriptions, was shown to restrain the lipopolysaccharide-induced expression of NF-κB, IL-1, and IL-6 (Shin et al., 2008).

## Delayed Onset of Diarrhea

Irinotecan is one of the most frequently used agents in advanced CRC patients (Bosch et al., 2016), while the delayed onset of diarrhea is one of the dose-limiting side effects of irinotecan. Approximately 16–22% patients administered with irinotecan alone experienced grade 3 or 4 diarrhea, which also occurred in 11–14% patients undergoing irinotecan-based combined regimens, including FORFIRI (Stein et al., 2010). The delayed onset of diarrhea is related to the metabolism of irinotecan. Irinotecan can be metabolized to its active metabolite (SN-38) in the liver, which is then conjugated with the uridine diphosphate glucuronosyltransferase to form SN-38 glucuronide, its inactive form. SN-38 glucuronide can be de-conjugated by the bacterial β-glucuronidase when it is excreted to the intestinal lumen through bile; afterwards, it is transformed to its active form, SN-38, again in the intestinal tract (Yamamoto et al., 1994). SN-38 accumulation in the intestinal tract will induce mucosal damage, resulting in the delayed onset of diarrhea (Atsumi et al., 1995; Takasuna et al., 1996). Various existing methods have been discovered to alleviate diarrhea. For instance, loperamide, an opioid receptor agonist, has been extensively used as an adjuvant for relieving irinotecan-induced diarrhea. Nonetheless, almost all of the treatments often become difficult in patients with severe diarrhea, and no satisfactory outcome has been achieved yet (Kon et al., 2018). Further, no reliable preventive methods have been established so far (Ikarashi et al., 2011).

### Banxia Xiexin Decoction

Banxia Xiexin Decoction, a prescription effective in relieving the above-mentioned oral mucositis, is also recognized to slow down the enterohepatic circulation of SN-38. On this account, Banxia Xiexin Decoction has been illustrated to exhibit protective effects against irinotecan-caused intestinal toxicity, which was achieved through inhibiting β-glucuronidase activity and prostaglandin E2 synthesis (Takasuna et al., 1995; Kase et al., 1997). Moreover, Banxia Xiexin Decoction was also found to outstandingly delay the onset of diarrhea in patients treated with cisplatin and irinotecan in a randomized controlled trial (RCT) (Mori et al., 2003). Another study demonstrated that Banxia Xiexin Decoction could reduce the frequency of severe diarrhea (grade 3 or 4) (Komatsu et al., 2010). Together, Banxia Xiexin Decoction turned out to be a reliable treatment with satisfactory outcomes in relieving the delayed-onset of diarrhea in clinic and was demonstrated to target certain enzyme in the process of irinotecan metabolism in preclinical studies, making it a potent formula for diarrhea treatment in clinical practice.

### Shengjiang Xiexin Decoction

Shengjiang Xiexin Decoction, a CHM prescription derived from Banxia Xiexin Decoction, contains one additional herb: *Zingiberis Rhizoma Recens*. Used to manage gastrointestinal disorders, Shengjiang Xiexin Decoction has been demonstrated to mitigate irinotecan-induced delayed onset of diarrhea in rats through inhibiting intestinal cell apoptosis, as well as triggering intestinal injury repair by promoting intestinal stem cell proliferation and inhibiting β-glucuronidase activity which is a key factor in accumulating SN-38, the toxic irinotecan metabolite (Deng et al., 2017). However, Shengjiang Xiexin Decoction was also reported to alter the pharmacokinetics of irinotecan by down-regulating the key factors involved in irinotecan metabolism (including hepatic multidrug resistance-associated protein-2) and P-glycoprotein expression, as well as changing the activities of carboxylesterase 2 and uridine diphosphate-glucuronosyltransferase 1A1 (Guan et al., 2017). So far, no experiment has demonstrated that the pharmacokinetic alterations of irinotecan caused by Shengjiang Xiexin Decoction will reduce its anti-tumor activity.

### Huangqin Decoction

Huangqin Decoction, or PHY906, is a prescription made up of *Scutellaria Baicalensis Radix, Glycyrrhizae Radix, Radix Paeoniae Alba*, and *Jujubae Fructus*. It is frequently used to manage different gastrointestinal symptoms, such as diarrhea, nausea and vomiting. In a phase 1/2 double-blind RCT, Huangqin Decoction was discovered to remarkably alleviate gastrointestinal toxicity, especially nausea and diarrhea in metastatic CRC patients undergoing irinotecan, 5-Fu, and LV (5-Fu/LV) regimens (Farrell and Kummar, 2003; Kummar et al., 2011). Meanwhile, it can enhance chemotherapeutic efficacy without changing the pharmacokinetics of irinotecan and 5-Fu/LV (Lam et al., 2015). More importantly, Huangqin Decoction was shown to restore intestinal epithelial damage through enhancing intestinal progenitor or stem cell regeneration, while simultaneously inducing anti-inflammatory activity in mice by down-regulating the expression of TNF-α, NF-κB, and COX-2 in neutrophils and macrophages. Additionally, it can also potentiate Wnt3a activity in human embryonic kidney-293 cells *in vitro* (Lam et al., 2010).

## Intestinal Mucositis

Intestinal mucositis is one of the most frequently encountered side effects in CRC patients undergoing chemotherapy regimens, such as 5-Fu and irinotecan. 50%-80% of patients developed intestinal mucositis when undergoing 5-Fu-based chemotherapy (Longley et al., 2003; Smith et al., 2008). This chemotherapy regimen, which can directly damage the mucosa cells through up-regulating pro-apoptosis, also plays a vital role in activating pro-inflammatory cytokines (like TNF-α) and triggering the NF-κB-mediated ceramide and caspase pathways, ultimately leading to excessive production of TNF-α, IL-1 β, and IL-6 and thus cause tissue damage (Chang et al., 2012).

### Chimonanthus nitens var. salicifolius

*Chimonanthus nitens var. salicifolius* (CS, Lamei in Chinese) is an herb for treating enteral disease. It was demonstrated in a 5-Fu-induced mucositis mouse model that the aqueous extract of CS can alleviate gastrointestinal mucositis. Moreover, experimental results have indicated that the aqueous extract of CS can lead to weight gain in mice, alleviating diarrhea and hemafecia, maintaining the intestinal length, reducing villus shortening, and inhibiting apoptosis and inflammation in the small intestine (Liu et al., 2013b). These findings demonstrate that CS exerts certain protective effect on chemotherapy-caused intestinal mucositis, which may be attributed to down-regulated apoptosis and inflammation.

### Pianzaihuang

The main ingredients of Pianzaihuang include *Moschus, Calculus Bovis*, Snake Gall, and *Radix Notoginseng*. It is a popular prescription that has been demonstrated to be effective in managing 5-Fu-induced intestinal mucositis in the CT-26 xenograft colon cancer-bearing mouse model. The diarrhea score was decreased in Pianzaihuang treated mice and the intestinal crypt damage in the colon was also restored in those mice, through inhibiting the expression of apoptotic proteins (Fu et al., 2017).

### Buzhong Yiqi Decoction

Buzhong Yiqi Decoction is composed of 8 herbs, including *Astragali Radix, Atractylodes Macrocephala, Cimicifugae Rhizoma, Citri Reticulatae Pericarpium, Bupleuri Radix, Panax Ginseng, Glycyrrhizae Radix*, and *Radix Angelicae Sinensis*. It has been widely adopted clinically and demonstrated to be effective as an adjuvant treatment for neoplasms, in which it could increase food intake, mitigate side effects, alleviate the cancer-related fatigue, and improve the quality of life in post-surgery patients as well as those undergoing chemotherapy (Jeong et al., 2010; Chao et al., 2014). It has been further proved that Buzhong Yiqi Decoction was able to restore villi shortening, crypt destruction, reduce apoptosis and necrosis, alleviate neutrophil infiltration in intestinal mucosal epithelia, and inhibit up-regulated inflammatory factors, such as TNF-α and IL-1β. These results revealed that Buzhong Yiqi Decoction can inhibit 5-Fu-induced intestinal mucositis, which may be attained through suppressing the up-regulation of inflammatory cytokines, reducing apoptosis and necrosis in intestinal mucosal epithelia (Gou et al., 2016).

### TJ-114

TJ-114 is a combination of Xiaochaihu Decoction and Wulingsan Powder composing of *Bupleuri Radix, Pinelliae Tuber, Alismatis Rhizome, Scutellariae Radix, Ginseng Radix, Zizyphi Fructus, Poria, Polyporus, Atractylodis Lanceae Rhizome, Cinnamomi Cortex, Glycyrrhizae Radix*, and *Zingiberis Rhizoma*. It is a prescription popularly used in some Asian countries to treat intestinal disorders, including diarrhea and hemafecia. TJ-114 aqueous extracts gavage to xenograft colon cancer-bearing mice twice daily relieved 5-Fu-induced intestinal mucositis. It lowered the diarrhea score and alleviated villi shortening and crypt destruction compared with the vehicle control. Typically, it can inhibit apoptosis and lower the secretion of pro-inflammatory cytokines, such as TNF-α and IL-1β, thus eliciting the protective effect against chemotherapy-induced intestinal mucositis (Coates et al., 1983). Recent studies further confirmed that TJ-114 inhibited cytokine-mediated apoptosis in intestinal crypt cells (Kato et al., 2015).

## Nausea and Vomiting

Nausea and vomiting are the most commonly experienced side effects in patients receiving chemotherapy. Briefly, chemotherapy can trigger serotonin secretion from the enterochromaffin cells lining the gastrointestinal tract. Serotonin can then stimulate the type-3 vagal afferent serotonin receptors (5-HT3) located in the gastrointestinal tract, as well as the nucleus tractus of the medulla oblongata and the chemoreceptor trigger zone located outside the blood-brain barrier. Serotonin can also send impulses to the vomiting center upon stimulation by an emetogenic substance P (Janelsins et al., 2013). Typically, blocking 5-HT3, or substance P is an effective method to prevent chemotherapy-induced nausea and vomiting (CINV). Currently, ondansetron, granisetron, and dolasetron are the alternative choices with satisfactory outcomes (Boussios et al., 2012) (Boussios et al., 2012). However, patients undergoing chemotherapy may still experience emesis. Undoubtedly, other treatments like CHMs are needed for a better management of CINV.

### Liujunzi Decoction

Liujunzi Decoction is composed of *Codonopsis Pilosula, Poria Cocos, Atractylodes Macrocephala, Pinellia Ternata, Citri Reticulatae Pericarpium*, and *Glycyrrhiza Radix*. Animal experiments have demonstrated that Liujunzi Decoction can antagonize the 5-HT3 receptor (Tominaga et al., 2011). Furthermore, it can also antagonize the 5-hydroxytryptamine receptor 2B and 5-hydroxytryptamine receptor 2C to avoid the cisplatin-induced reduction in ghrelin levels, thereby resulting in the recovery of food intake (Takeda et al., 2008). In addition, Liujunzi Decoction was also shown to alleviate CNIV in patients undergoing cisplatin and paclitaxel regimen (Ohnishi et al., 2017).

### Ginger

Ginger is the most extensively applied supplement for preventing and/or reducing CINV. It has long been used by practitioners in the management of gastrointestinal discomforts, such as nausea and excessive flatulence (Shukla and Singh, 2007). As shown in a clinical study, 3 different daily doses of ginger (namely 0.5, 1.0, and 1.5 g) combined with standard 5-HT3 receptor antagonists and dexamethasone can dramatically reduce acute CINV compared with the application of placebo in combination with the standard 5-HT3 receptor antagonists and dexamethasone (Ryan et al., 2009). However, it was also reported that ginger itself did not show a significant difference compared with standard 5-HT3 receptor antagonists or dexamethasone in patients undergoing chemotherapy in a systemic review (Ernst and Pittler, 2000).

Furthermore, other CHMs, such as *Poria cocos, Glycyrrhiza Radix*, and *Atractylodes Macrocephala*, as well as prepared medicines like Kanglaite Injection, Javanica oil emulsion injection, and Aidi injection, have also been reported to alleviate CINV, as illustrated in a systemic review (Chen et al., 2016). *Astragali Radix*, which has been mentioned above, was indicated in some studies that its compound decoction can reduce the proportion of patients developing nausea and vomiting, as well as the rate of leucopenia, in CRC patients undergoing chemotherapy (Wu et al., 2005).

## Conclusion

Even though investigators have been studying CHMs and chemotherapy-induced side effects for years, there is still growing interest in defining the mechanisms and effects of CHMs in preventing or attenuating chemotherapy-induced side effects. A summary of the main herbs and decoctions for treating chemotherapy-induced side effects is listed in Table 1. Current evidence from preclinical studies continually supports the notion that CHMs are effective in preventing or alleviating chemotherapy-induced side effects. More importantly, some of the reported CHMs have been identified to have no effect on the pharmacokinetics or anti-tumor effect of chemotherapeutic reagents when used together with chemotherapy in animal studies, clarifying that these CHMs might be combined with chemotherapy without reducing its efficacy. However, not all the results of clinical evaluation on the efficacies of CHMs in the management of chemotherapy-induced side effects are consistent with preclinical studies, such as ginger. This certainly could be due to several factors, including small sample size, low doses of CHMs, chemotherapeutic regimens that have moderate side effects and the lack of placebo control. Also, there is a lack of pharmacokinetics evaluation of chemotherapeutic drugs in patients treated with CHMs undergoing chemotherapy. In addition, only a few studies have evaluated the safety of CHMs themselves, although many of them have been applied clinically for centuries. Thus, it becomes imperative to conduct larger sample size, double-blinded, randomized, placebo-controlled clinical trials to provide stronger evidence to further confirm the effects and safety of CHMs in the management of chemotherapy-induced side effects. Moreover, it is critically important to further identify whether CHMs have effects on the metabolism of chemotherapeutic reagents and anti-tumor efficacy in clinical trial. It is also contended that more effort needs to be taken to identify the related signaling pathways involved in chemotherapy-related toxicity, which will ultimately improve the understanding of the role of CHMs in the management of chemotherapy-induced side effects.

**Table 1:** Summary of the effect of CHMs against chemotherapy-induced side effects.

**Types of side effect**	**Chinese herbal medicine (formulas or herbs)**	**Main ingredient/herbs**	**Evidence**
Neurotoxicity	*Astragali Radix* (Di Cesare Mannelli et al., 2015, 2017; Deng et al., 2016a)		Pre-clinical: Reduce oxaliplatin-induced cold hypersensitivity, block the onset of the pro-allodynia effect, and relieve neuro-damage-induced; Down-regulate the expression of the activating transcription factor 3 in the dorsal root ganglia.
	*Tumeric* (Al Moundhri et al., 2013; Mendonça et al., 2013; Sharma et al., 2014)	Curcumin	Pre-clinical: Reducing the high plasma neurotensin and platinum accumulation in the sciatic nerve.
	Guilong Tongluo Decoction (Liu et al., 2013a)	*Ramulus Cinnamomi, Astragali Radix*, Earthworm*, Carthamus Tinctorius, Radix Angelicae Sinensis, Ligusticum, Spatholobus, Radix Paeoniae Alba, Rhizoma Curcumae*, and *Glycyrrhizae Radix*.	Clinical: alleviate the development of grades 1-2 neurotoxicity after 6 cycles of adjuvant oxaliplatin-based chemotherapy in CRC patients, and delayed the onset time of grades 1-4 neurotoxicity.
	Niuche Shenqi Wan (Kono et al., 2011, 2013, 2015; Ushio et al., 2012)	*Rehmannia Viride Radix, Achyranthis Bidentatae Radix, Corni Fructus, Dioscorea Opposita Rhizoma, Plantaginis Semen, Alismatis Rhizoma, Moutan Cortex, Cinnamomi Cortex, Aconiti Lateralis Praeparata Tuber* and *Poria Alba*.	Pre-clinical: Increase peripheral blood flow, stimulate spinal kappa-opioid receptors, and inhibit oxidative stress or activation of the C fiber. Clinical: Decrease the incidence and delay the development of grade 2 and above oxaliplatin-induced neurotoxicity in CRC after 8 cycles of oxaliplatin-based chemotherapy.
	Wenluotong Decoction (Deng et al., 2016b; Noh et al., 2018)	*Epimedium Brevicornum Maxim, Geranium Wilfordii Maxim, Cinnamomum Cassia Presl* and *Carthamus Tinctorius*.	Pre-clinical: Reverse both glial activation in the spinal dorsal horn and nociceptive sensitization of oxaliplatin-resultant chronic neuropathic pain in rats.
	Guizhi Jiazhufu Decoction (Hosokawa et al., 2012)	*Cinamomi Cortex, Aconiti Lateralis Praeparata Tuber, Zingiberis Rhizoma, Jujubae Fructus, Glycyrrhizae Radix*, and *Atracylodis Macrocephalae Rhizoma*	Clinical: Reduce neuropathy in metastatic CRC patients undergoing the FOLFOX regimen.
	Shaoyao Gancao Decoction (Hosokawa et al., 2012; Andoh et al., 2017)	*Paeonia Alba Radix* and *Glycyrrhizae Radix*.	Pre-clinical: Suppress transient receptor melastatin 8 mRNA expression in the mouse dorsal root ganglia. Clinical: Response rate was reported to be 65% in advanced CRC patients with oxaliplatin-related neuropathy.
	Huangqi Guizhi Wuwu Decoction (Jun et al., 2013; Cheng et al., 2017)	*Astragali Radix, Cinnamomi Cortex, Radix Paeoniae Alba, Zingiberis Rhizoma* and *Jujubae Fructus*.	Pre-clinical: Down-regulate inflammation and immune responses. Clinical: Lower the incidence and relieve the severity of neurotoxicity in patients undergoing the FOLFOX regimen.
Oral mucositis	Banxia Xiexin Decoction (Matsuda et al., 2015; Nishikawa et al., 2018)	*Pinellia Ternata, Scutellaria Baicalensis Radix, Panax Ginseng, Rhizoma Coptidis, Glycyrrhizae Radix, Zingiberis Rhizome and Jujubae Fructus*.	Clinical: Reduce the severity grade (≥2) and the duration of chemotherapy-induced oral mucositis in gastric cancer and CRC patients.
	*Lonicerae Flos* (Schröder et al., 2013)		Pre-clinical: Suppress the expression of COX-2, IL-1 and IL-6.
Delayed onset of diarrhea	Banxia Xiexin Decoction (Takasuna et al., 1995; Kase et al., 1997; Mori et al., 2003; Komatsu et al., 2010)	*As described above*.	Pre-clinical: Inhibit β-glucuronidase activity and prostaglandin E2 synthesis. Clinical: Delay the onset of diarrhea in patients treated with cisplatin and irinotecan. Reduce the frequency of severe diarrhea (grade 3 or 4).
	Shengjiang Xiexin Decoction (Deng et al., 2017; Guan et al., 2017)	*Pinellia Ternata, Scutellaria Baicalensis Radix, Panax Ginseng, Rhizoma Coptidis, Glycyrrhizae Radix, Zingiberis Rhizome, Zingiberis Rhizoma Recens and Jujubae Fructus*.	Pre-clinical: Inhibit intestinal cell apoptosis, as well as triggering intestinal injury repair by promoting intestinal stem cell proliferation and inhibiting β-glucuronidase activity.
	Huangqin Decoction (Farrell and Kummar, 2003; Lam et al., 2010, 2015; Kummar et al., 2011)	*Scutellaria Baicalensis Radix, Glycyrrhizae Radix, Radix Paeoniae Alba* and *Jujubae Fructus*.	Pre-clinical: Restore intestinal epithelial damage through enhancing intestinal progenitor or stem cell regeneration; Induce anti-inflammatory activity in mice by down-regulating the expression of TNF-α, NF-κB and COX-2 in neutrophils and macrophages; potentiate Wnt3a activity.
			Clinical: Alleviate nausea and diarrhea in metastatic CRC patients undergoing irinotecan, 5-Fu, and LV (5-Fu/LV) regimens.
Intestinal mucositis	*Chimonanthus nitens var. salicifolius* (Liu et al., 2013b)		Pre-clinical: Alleviate 5-Fu induced diarrhea and hemafecia, maintain the intestinal length, reduce villus shortening, and inhibit apoptosis and inflammation in the small intestine.
	Pianzaihuang (Fu et al., 2017)	*Moschus,Calculus Bovis*, Snake Gall *and Radix Notoginseng*.	Pre-clinical: Reduce 5-Fu-induced intestinal crypt damage through inhibiting the expression of apoptotic proteins.
	Buzhong Yiqi Decoction (Jeong et al., 2010; Chao et al., 2014; Gou et al., 2016)	*Astragali Radix, Atractylodes Macrocephala, Cimicifugae Rhizoma, Citri Reticulatae Pericarpium, Bupleuri Radix, Panax Ginseng, Glycyrrhizae Radix* and *Radix Angelicae Sinensis*.	Pre-clinical: Restore villi shortening, crypt destruction, reduce apoptosis and necrosis, alleviate neutrophil infiltration in intestinal mucosal epithelia, and inhibit up-regulated inflammatory factors, such as TNF-α and IL-1β.
	TJ-114 (Coates et al., 1983; Kato et al., 2015)	*Bupleuri Radix, Pinelliae Tuber, Alismatis Rhizome, Scutellariae Radix, Ginseng Radix, Zizyphi Fructus, Poria, Polyporus, Atractylodis Lanceae Rhizome, Cinnamomi Cortex, Glycyrrhizae Radix*, and *Zingiberis Rhizoma*.	Pre-clinical: Lower the diarrhea score and alleviated villi shortening and crypt destruction. Also, it can inhibit apoptosis and lower the secretion of pro-inflammatory cytokines, such as TNF-α and IL-1β.
Nausea and vomiting	Liujunzi Decoction (Takeda et al., 2008; Tominaga et al., 2011; Ohnishi et al., 2017)	*Codonopsis Pilosula, Poria Cocos, Atractylodes Macrocephala, Pinellia Ternata, Citri Reticulatae Pericarpium* and *Glycyrrhiza Radix*.	Pre-clinical: Antagonize the 5-HT3 receptor, the 5-hydroxytryptamine receptor 2B and 5-hydroxytryptamine receptor 2C to avoid the cisplatin-induced reduction in ghrelin levels. Clinical: Alleviate CNIV in patients undergoing cisplatin and paclitaxel regimen.
	Ginger (Ernst and Pittler, 2000; Shukla and Singh, 2007; Ryan et al., 2009)		Clinical: Ginger combined with standard 5-HT3 receptor antagonists and dexamethasone can reduce acute CINV compared with the application of placebo in combination with the standard 5-HT3 receptor antagonists and dexamethasone.

## Author Contributions

WC provided the idea and designed the theoretical framework. DC and JZ searched literature. WC and DC wrote the article.

### Conflict of Interest Statement

The authors declare that the research was conducted in the absence of any commercial or financial relationships that could be construed as a potential conflict of interest.
